# Supplementary figures and images for: Deep sequencing of yeast and mouse tRNAs and tRNA fragments using OTTR
Source: eLife. 2025 Apr 25;14:e77616. doi: 10.7554/eLife.77616 (PMC12140624; doi:10.7554/eLife.77616)

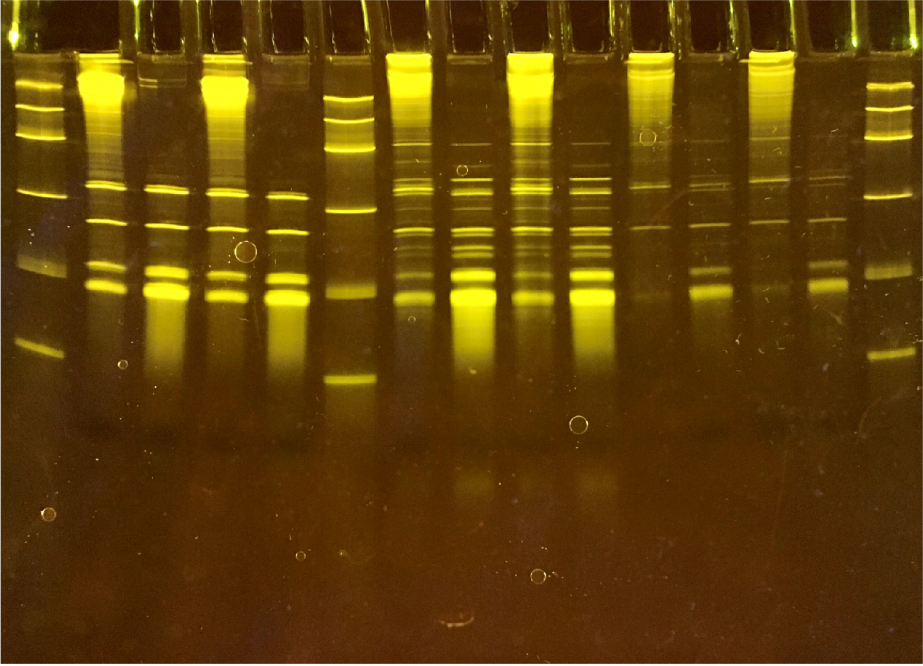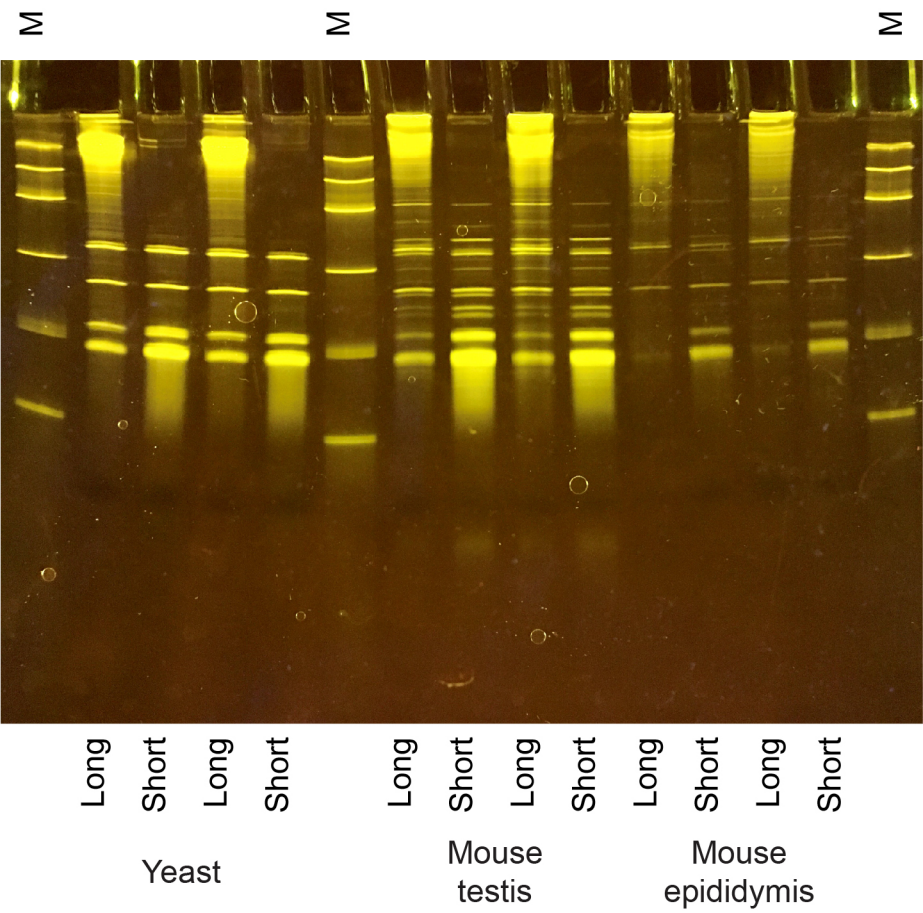

Supplement: Figure 1—figure supplement 1—source data 1. [file elife-77616-fig1-figsupp1-data1.pdf]

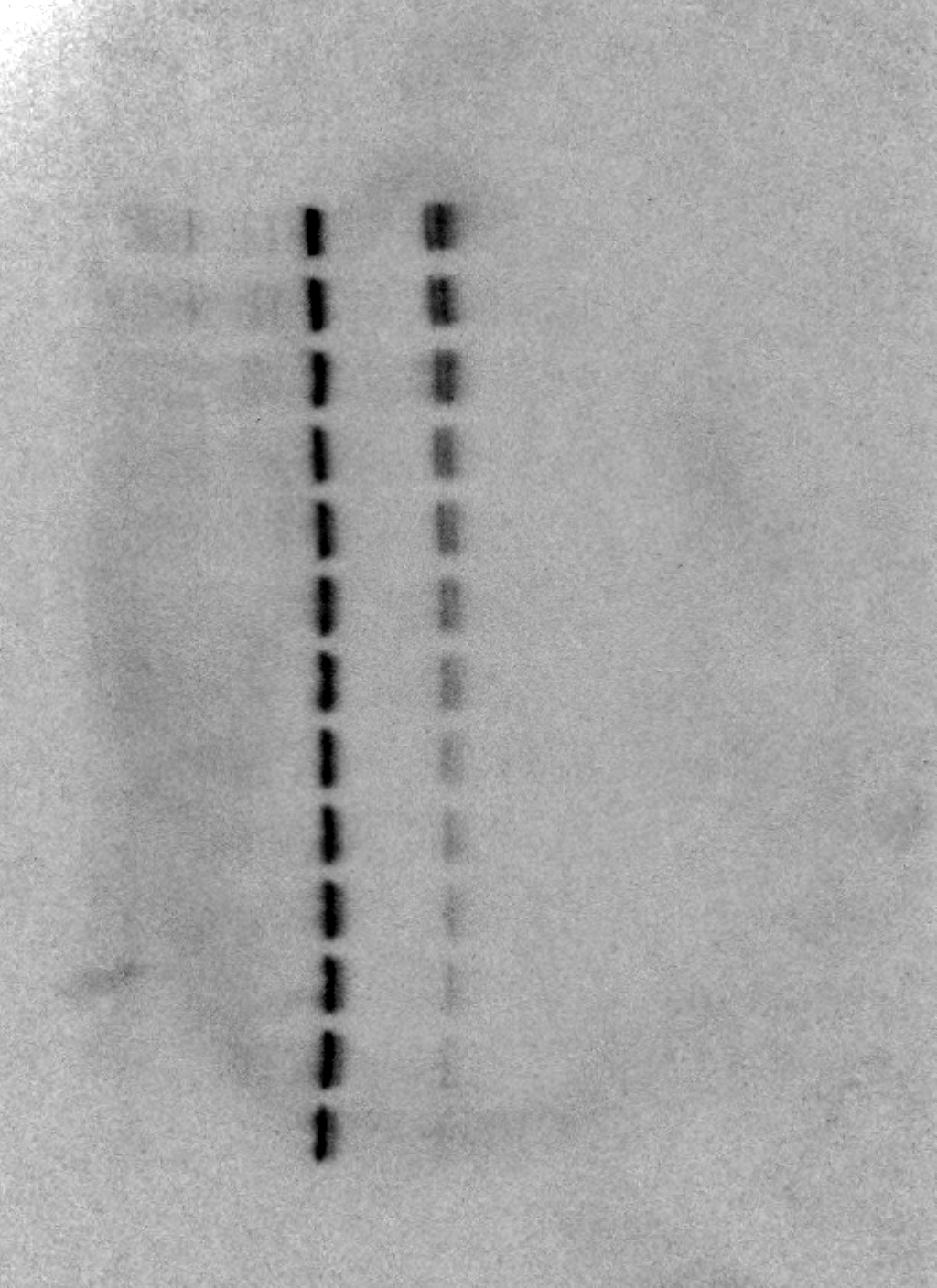

Supplement: Figure 3—source data 1. [file elife-77616-fig3-data1.zip › Gustafsson_Figure_3-source_data_1.tif]

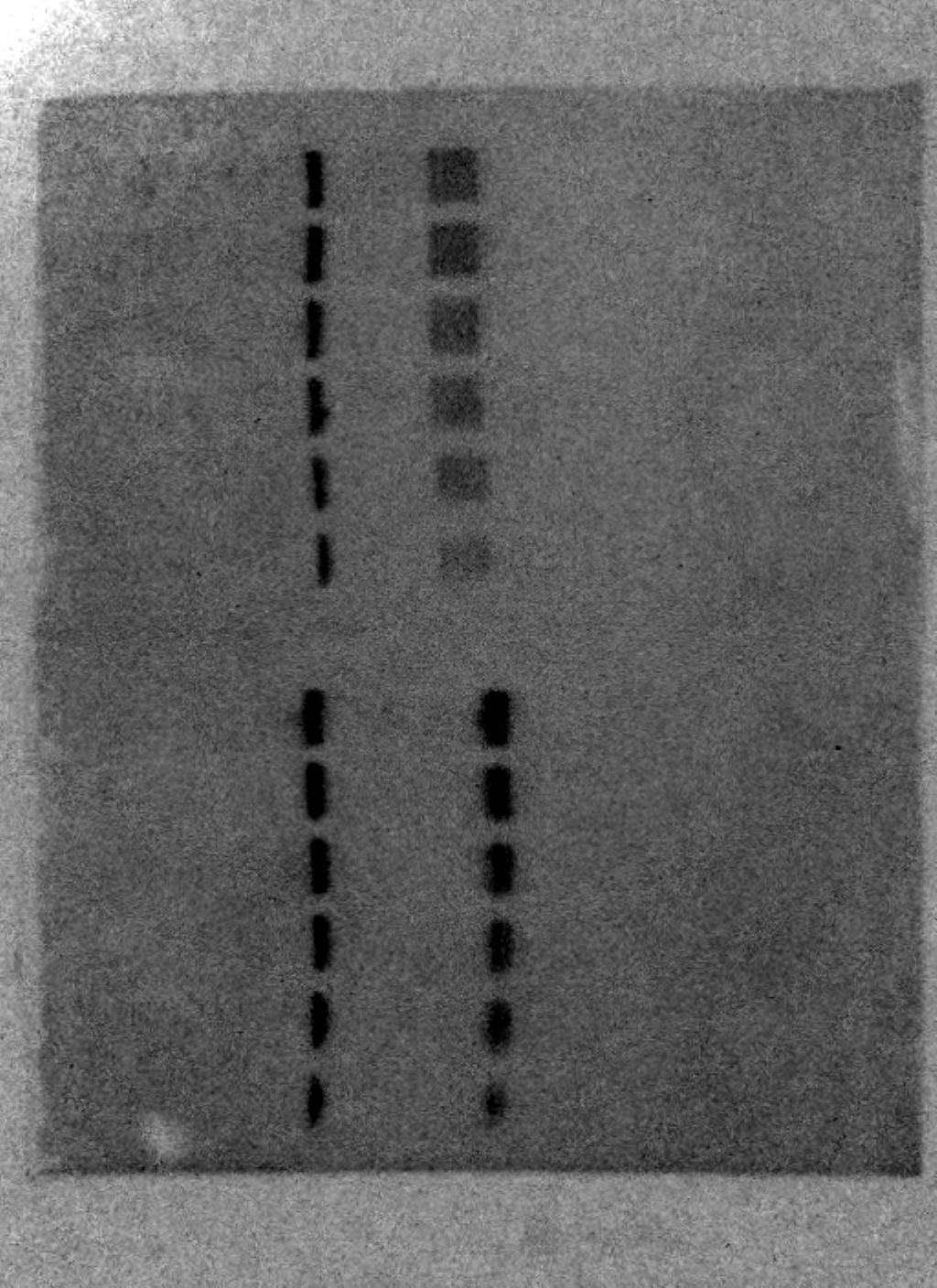

Supplement: Figure 3—source data 1. [file elife-77616-fig3-data1.zip › Gustafsson_Figure_3-source_data_2.tif]

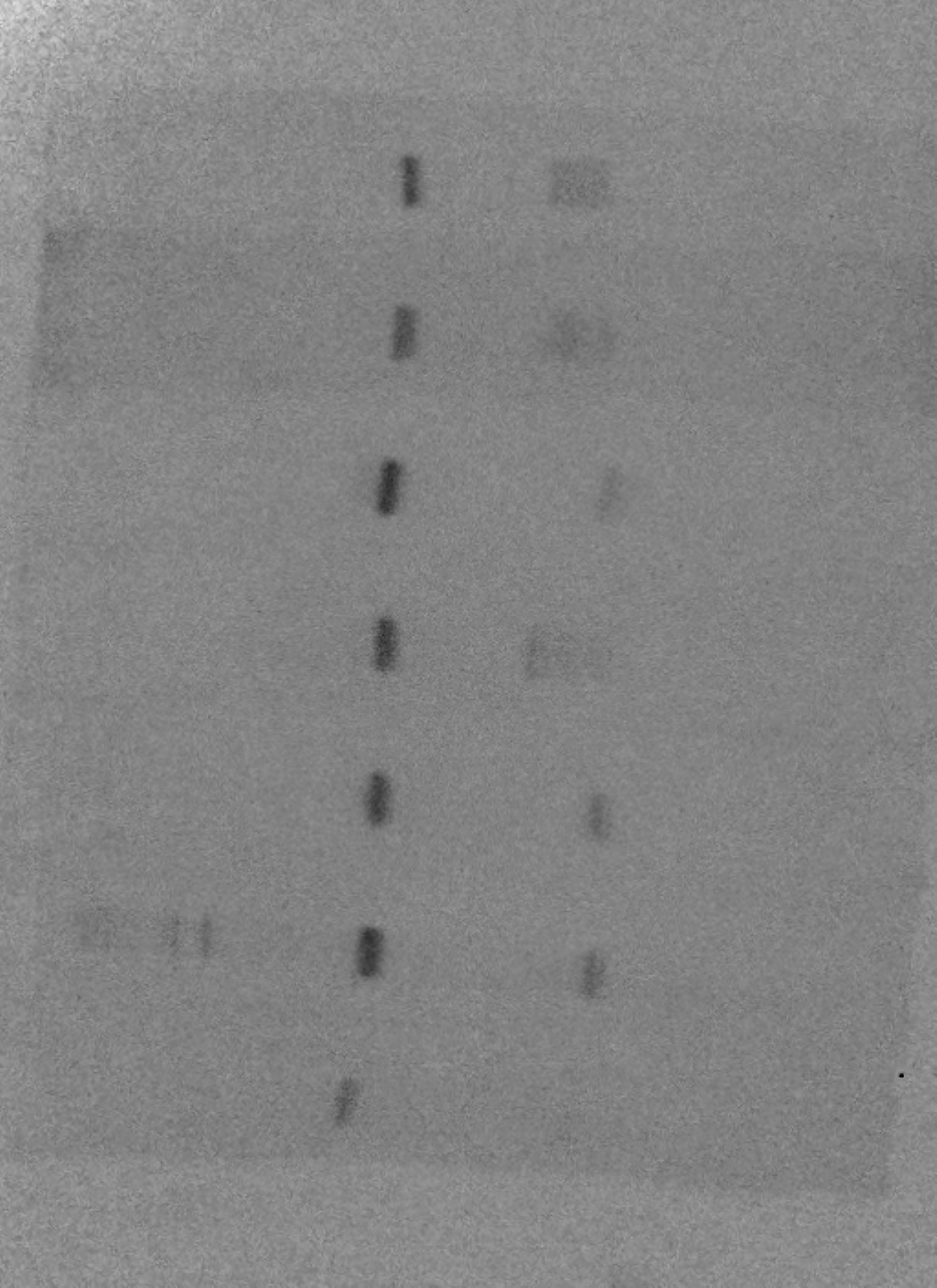

Supplement: Figure 3—source data 1. [file elife-77616-fig3-data1.zip › Gustafsson_Figure_3-source_data_3.tif]

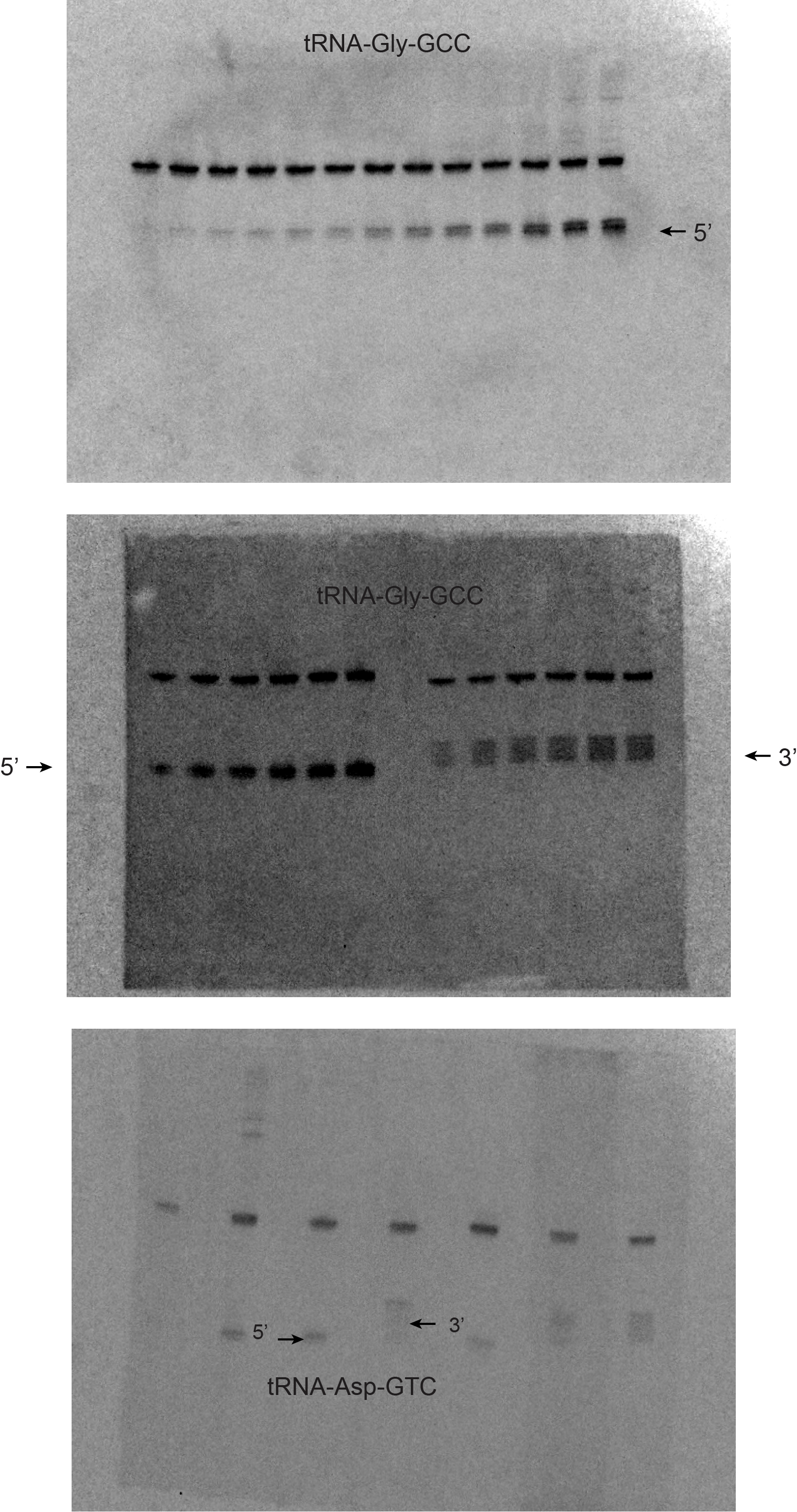

Supplement: Figure 3—source data 2. [file elife-77616-fig3-data2.zip › Gustafsson_Figure_3-source_data_annotated.jpg]

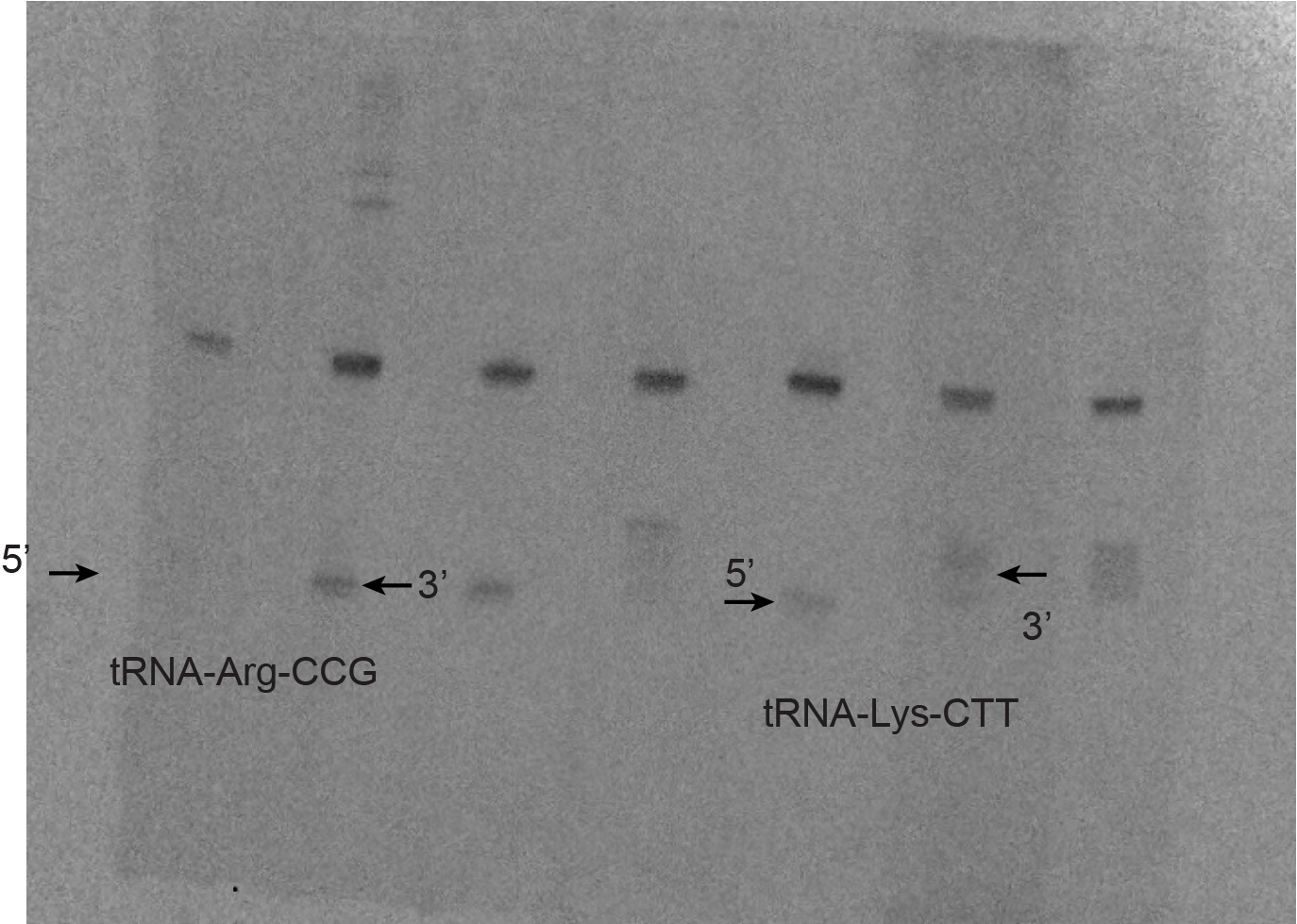

Supplement: Figure 3—figure supplement 2—source data 2. [file elife-77616-fig3-figsupp2-data2.zip › Gustafsson_Figure_S5-source_data_annotated.jpg]
